# Exploratory analysis using machine learning of predictive factors for falls in type 2 diabetes

**DOI:** 10.1038/s41598-022-15224-4

**Published:** 2022-07-13

**Authors:** Yasuhiro Suzuki, Hiroaki Suzuki, Tatsuya Ishikawa, Yasunori Yamada, Shigeru Yatoh, Yoko Sugano, Hitoshi Iwasaki, Motohiro Sekiya, Naoya Yahagi, Yasushi Hada, Hitoshi Shimano

**Affiliations:** 1 Department of Rehabilitation Medicine, University of Tsukuba Hospital, Tsukuba, Ibaraki 305-8576 Japan; 2 Department of Internal Medicine (Endocrinology and Metabolism), Faculty of Medicine, University of Tsukuba, Tsukuba, Ibaraki 305-8575 Japan; 3 IBM Research, Tokyo, 103-8510, Japan; 4 International Institute for Integrative Sleep Medicine (WPI-IIIS), University of Tsukuba, Tsukuba, Ibaraki 305-8575 Japan; 5 Life Science Center of Tsukuba Advanced Research Alliance (TARA), University of Tsukuba, Tsukuba, Ibaraki 305-8577 Japan; 6 Japan Agency for Medical Research and Development-Core Research for Evolutional Science and Technology (AMED-CREST), Chiyoda-ku, Tokyo, 100-0004, Japan

**Keywords:** Endocrinology, Health care, Medical research

## Abstract

We aimed to investigate the status of falls and to identify important risk factors for falls in persons with type 2 diabetes (T2D) including the non-elderly. Participants were 316 persons with T2D who were assessed for medical history, laboratory data and physical capabilities during hospitalization and given a questionnaire on falls one year after discharge. Two different statistical models, logistic regression and random forest classifier, were used to identify the important predictors of falls. The response rate to the survey was 72%; of the 226 respondents, there were 129 males and 97 females (median age 62 years). The fall rate during the first year after discharge was 19%. Logistic regression revealed that knee extension strength, fasting C-peptide (F-CPR) level and dorsiflexion strength were independent predictors of falls. The random forest classifier placed grip strength, F-CPR, knee extension strength, dorsiflexion strength and proliferative diabetic retinopathy among the 5 most important variables for falls. Lower extremity muscle weakness, elevated F-CPR levels and reduced grip strength were shown to be important risk factors for falls in T2D. Analysis by random forest can identify new risk factors for falls in addition to logistic regression.

## Introduction

Approximately one-third of older adults experience falls each year, and, of the fallers, one-fourth are seriously injured^1^. Fall-related injuries among older adults often result in a bedridden condition. Even without a serious injury falls are independently associated with functional decline in older adults^2^. As a result, falls impose a substantial economic burden on individuals, society and the healthcare system^3,4^.

Major risk factors for falls are previous falls, balance impairment, decreased muscle strength, visual impairment, specific medications, gait disturbances and cognitive decline^5,6^. Diabetes is also a risk factor for falls^7^ with the risk in older adults with diabetes reported to be 1.5–3 times higher than in those without diabetes^7^. Previously reported risk factors for falls among those with diabetes include diabetic polyneuropathy, diabetic retinopathy, increased cystatin C levels and insulin or sulfonylurea use as well as decreased grip, knee extension and ankle dorsiflexion strength^8–12^. However, only a few studies have comprehensively analyzed the associations between those risk factors and falls in persons with diabetes^8,10,11^. In a prospective cohort study of risk factors for falls in older adults with diabetes, multiple logistic regression showed that A1C ≤ 6% in insulin users, weight loss during follow-up, increased diastolic blood pressure change during postural changes from sitting to standing, peripheral nerve dysfunction and poor standing balance were significant risk factors for falls^10^. Moreover, that study showed that decreased grip strength and decreased walking speed for the 6-m walk test were risk factors for falls but without significance; also knee extension strength was not included as an explanatory variable in the multiple logistic regression analysis^10^.

Despite our understanding of the seriousness of falls and the resultant high cost of medical care, identification of the few most critical factors that are strongly predictive of falls in high risk populations has been lacking, partly because over 400 factors have been linked with falls in adult populations^13^. Risk factors for falls are diverse and interact with each other. Not all contribute to falls to the same degree. Moreover, the risk may vary depending on the factors for which they are adjusted. To comprehensively evaluate risk factors for falls and to identify those with the highest contribution to falls are important because interventions to effectively prevent falls should be targeted to groups at the greatest risk. However, as the number of predictors increases, especially with a small number of study participants, traditional statistical models such as logistic regression might be unsuitable for identifying important variables as predictors^14,15^. A simulation study on logistic regression found that when the number of events per variable was less than 10, the regression coefficients were biased^15^. This association was also observed for stepwise logistic regression in another simulation study^14^. One of the more appropriate approaches is the nonlinear and nonparametric random forest algorithm, which is one type of machine learning based on the ensemble learning method^16^. In random forest a large number of decision trees are created using bootstrapped data, where a splitting variable at each node is selected from a randomly chosen subset of predictors. Final prediction is determined by a majority vote in the tree ensemble. By examining risk factors for falls using two different methods, logistic regression and random forest algorithm, the risks may be analyzed from different perspectives.

Individuals who use walking aids or have reduced walking ability and activities of daily living are at a very high risk of falls as their physical functions such as muscle strength, balance capability and cognition are already reduced. The goal of our study was to identify individuals with type 2 diabetes who are independent in activities of daily living but are at high risk for falls so that they can be provided with appropriate interventions to prevent falls. In this context, we collected data on muscle strength, balance capability, body composition, diabetic microvascular and macrovascular complications, laboratory examinations and medications and aimed to identify important predictors of falls in those with type 2 diabetes who have been independent in daily life using traditional logistic regression and random forest.

## Results

**Figure 1:**
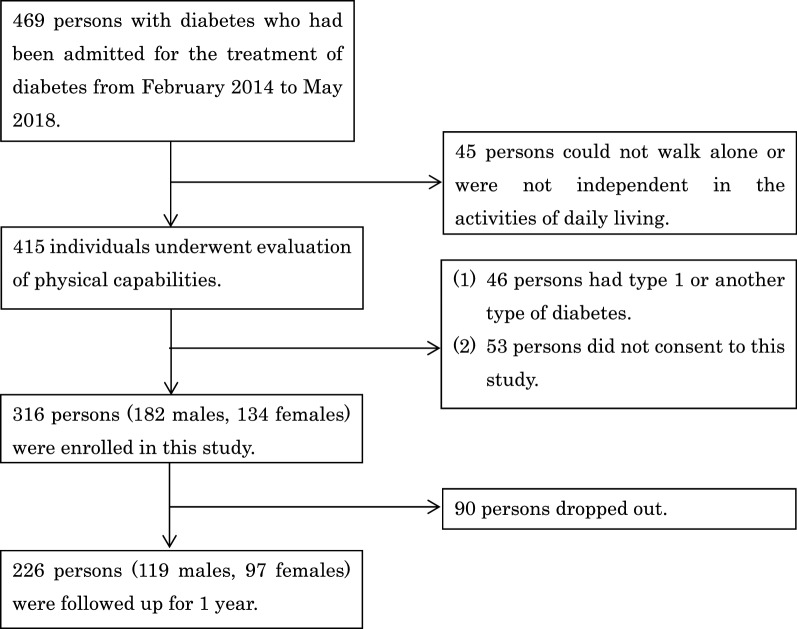
Flowchart of selection of study participants.

The collection rate of the questionnaire was 72% (226/316, 129 men and 97 women, median age 62 years (49, 68) (Fig. 1). There were no missing data. Forty-four of the 226 participants had a fall in the year after hospital discharge, for a fall rate of 19%.

The faller group had a significantly higher rate of females, impaired vibratory sensation, proliferative retinopathy and history of stroke and higher levels of fasting C-peptide (F-CPR) compared with the non-faller group (Table 1). However, age, duration of diabetes, DPN and nephropathy were not significantly different between the two groups (Table 1). Although medications both on admission and at discharge were not significantly different between groups, the proportion of sulfonylurea users tended to be higher in the faller group than in the non-faller group (Table 2).

**Table 1:** Characteristics of participants.

	All *n* = 226	Fallers *n* = 44	Non-fallers *n* = 182	*P*
Age (y)	62 (49, 68)	63 (54, 69)	62 (48, 68)	0.254
Female sex, n (%)	97 (42.9)	26 (59.0)	71 (39.0)	0.016
Duration of diabetes (y)	10 (2, 16)	11 (3, 20)	9 (2, 15)	0.070
Height (m)	1.61 (1.55, 1.68)	1.60 (1.53, 1.65)	1.62 (1.55, 1.70)	0.171
Body weight (kg)	69.8 ± 17.7	70.7 ± 19.0	69.6 ± 17.3	0.692
Body mass index (kg/m^2^)	26.5 ± 5.6	27.4 ± 6.0	26.3 ± 5.4	0.281
Fasting plasma glucose (mmol/l)	8.9 (7.3, 10.9)	9.4 (7.8, 11.1)	8.9 (7.2, 10.8)	0.279
Fasting serum C-peptide (nmol/l)	0.5 (0.4, 0.8)	0.7 (0.4, 0.9)	0.5 (0.3, 0.7)	0.032
HbA1c (%)	9.7 ± 1.9	9.5 ± 1.8	9.8 ± 1.9	0.399
HbA1c (mmol/mol)	83 ± 20	80 ± 20	83 ± 21	
Total cholesterol (mmol/l)	4.8 ± 1.1	4.7 ± 0.8	4.8 ± 1.1	0.408
LDL-cholesterol (mmol/l)	2.8 ± 0.8	2.8 ± 0.7	2.8 ± 0.8	0.788
HDL-cholesterol (mmol/l)	1.2 (1.0, 1.4)	1.2 (1.0, 1.4)	1.2 (1.0, 1.4)	0.682
Triglycerides (mmol/l)	1.4 (1.0, 2.0)	1.3 (1.1, 2.0)	1.5 (1.0, 2.0)	0.487
Creatinine (µmol/l)	68.2 ± 43.4	85.9 ± 75.6	63.9 ± 29.8	0.064
Estimated glomerular filtration rate (ml/min/1.73 m^2^)	83 (66, 99)	73 (53, 93)	85 (70, 101)	0.085
Urinary albumin excretion (mg/day)	10.4 (5.2, 31.6)	13.6 (6.0, 39.6)	10.2 (5.1, 25.7)	0.692
Diabetic polyneuropathy, n (%)	64 (28.3)	16 (36.4)	48 (26.4)	0.187
Impaired vibratory sensation, n (%)	53 (23.5)	16 (36.4)	37 (20.3)	0.024
Cardiac autonomic neuropathy, n (%)	83 (36.7)	19 (43.2)	64 (35.2)	0.322
Nephropathy, n (%)	66 (29.2)	16 (32.4)	50 (27.5)	0.244
Retinopathy, n (%)	16 (7.1)	7 (15.9)	9 (4.9)	0.011
baPWV (cm/s)	1636 ± 354	1656 ± 363	1631 ± 353	0.680
Coronary artery disease, n (%)	16 (7.1)	2 (4.5)	14 (7.7)	0.465
Stroke, n (%)	12 (5.3)	5 (11.4)	7 (3.8)	0.046
Peripheral arterial disease, n (%)	3 (1.3)	1 (2.3)	2 (1.1)	0.538

Data are mean ± SD or median (25th, 75th). LDL, low-density lipoprotein; HDL, high-density lipoprotein; baPWV, brachial-ankle pulse wave velocity.

**Table 2:** Medications administered to study participants.

	All	Fallers	Non-fallers	*P*
**Medications on admission, n (%)**				
Sulfonylureas	54 (23.9)	12 (27.3)	42 (23.1)	0.558
Glinides	6 (2.7)	2 (4.5)	4 (2.2)	0.385
Biguanides	118 (52.2)	22 (50.0)	96 (52.7)	0.743
SGLT2 inhibitors	23 (10.2)	5 (11.4)	18 (9.9)	0.772
α-glucosidase inhibitors	22 (9.7)	5 (11.4)	17 (9.3)	0.685
Thiazolidinediones	12 (5.3)	4 (9.1)	8 (4.4)	0.213
DPP-4 inhibitors	112 (49.6)	25 (56.8)	87 (47.8)	0.283
GLP-1 receptor agonists	12 (5.3)	4 (9.1)	8 (4.4)	0.213
Insulin	111 (49.1)	20 (45.5)	91 (50.0)	0.588
Antihyperlipidemic agents	90 (39.8)	17 (38.6)	73 (40.1)	0.858
Antihypertensive agents	88 (38.9)	20 (45.5)	68 (37.4)	0.323
**Medications at discharge, n (%)**				
Sulfonylureas	33 (14.6)	10 (22.7)	23 (12.6)	0.089
Glinides	6 (2.7)	0 (0)	6 (3.3)	0.222
Biguanides	150 (66.4)	28 (63.6)	122 (67.0)	0.669
SGLT2 inhibitors	21 (9.3)	3 (6.8)	18 (9.9)	0.529
α-glucosidase inhibitors	20 (8.8)	4 (9.1)	16 (8.8)	0.950
Thiazolidinediones	15 (6.6)	4 (9.1)	11 (6.0)	0.466
DPP-4 inhibitors	109 (48.2)	23 (52.3)	86 (47.3)	0.550
GLP-1 receptor agonists	24 (10.6)	7 (15.9)	17 (9.3)	0.204
Insulin	105 (46.5)	17 (38.6)	88 (48.4)	0.246
Antihyperlipidemic agents	90 (39.8)	17 (38.6)	73 (40.1)	0.858
Antihypertensive agents	80 (35.4)	18 (40.9)	62 (34.1)	0.394

SGTL2, sodium-glucose co-transporter 2; DPP-4, dipeptidyl peptidase-4; GLP-1, glucagon-like peptide-1.

Physical activity during the hospitalization period, body composition and physical capabilities of participants are shown in Table 3. Physical activities did not differ significantly between the faller group and the non-faller group. Skeletal muscle percentage in the faller group was significantly lower than in the non-faller group. On the other hand, skeletal muscle mass index (SMI) and body fat percentage were not significantly different between groups. Variables related to muscle strength including knee extension strength, knee extension endurance, dorsiflexion strength of the ankle joint, toe pinch force and grip strength in the faller group were significantly inferior to those in the non-faller group. For balance capabilities, IPS levels in the faller group were significantly lower than in the non-faller group. Other balance capability-related variables, including mIPS and one-leg standing time, tended to have lower values in the faller group.

**Table 3:** Physical activity, body composition and physical capabilities of participants.

	All	Fallers	Non-fallers	*P*
Physical activity				
Steps (steps/d)	7002 ± 3662	7593 ± 5510	6859 ± 3057	0.398
MVPA time (min/d)	13.9 (7.5, 26.0)	11.4 (6.1, 24.4)	14.4 (7.7, 26.0)	0.313
Body composition				
Skeletal muscle percentage (%)	37 ± 5	36 ± 5	38 ± 5	0.013
Body fat percentage (%)	33 ± 9	35 ± 9	32 ± 9	0.058
Skeletal muscle mass index (kg/m^2^)	8.3 ± 1.4	8.1 ± 1.4	8.3 ± 1.4	0.379
Muscle strength				
Knee extension strength (Nm/kg)	1.49 ± 0.47	1.22 ± 0.38	1.56 ± 0.46	< 0.001
Knee extension endurance (J)	826 (622, 1120)	719 (557, 874)	860 (638, 1138)	0.015
Dorsiflexion strength (ankle joint) (kgf)	32 ± 8	29 ± 7	33 ± 8	0.001
Toe pinch force (kgf)	3.6 (2.8, 4.6)	3.1 (2.3, 3.9)	3.7 (3.0, 4.7)	0.002
Grip strength (kgf)	29 (22, 37)	23 (20, 30)	30 (24, 38)	< 0.001
Balance capability				
Index of postural stability	1.62 (1.37, 1.82)	1.54 (1.28, 1.77)	1.63 (1.39, 1.84)	0.085
Modified index of postural stability	0.17 (0.07, 0.34)	0.15 (0.06, 0.23)	0.18 (0.07, 0.35)	0.187
One-leg standing time (s)	45 (15, 111)	26 (10, 79)	49 (18, 120)	0.052
Flexibility				
Finger-to-floor distance (cm)	–2 (–12, 6)	0 (–9, 8)	–3 (–12, 6)	0.175

Data are the mean ± SD or median (25th, 75th). MVPA, moderate-to-vigorous physical activity.

We performed logistic regression and a random forest classifier with sex, F-CPR level, impaired vibratory sensation, proliferative retinopathy, past history of stroke, taking sulfonylureas at discharge, skeletal muscle percentage, body fat percentage, knee extension strength, knee extension endurance, dorsiflexion strength, toe pinch force, grip strength, IPS and one-leg standing time as covariates in order to identify important risk factors for falls. Logistic regression showed that knee extension strength, F-CPR level and dorsiflexion strength were independent predictors of falls (Table 4). In the random forest, although the selected features changed somewhat in accordance with the training dataset, grip strength, F-CPR levels, knee extension strength, dorsiflexion strength and proliferative retinopathy were the 5 most important variables related to falls selected consistently multiplied by 10 and with tenfold cross validation (Table 5). During 10 random forest runs, explanatory variables were selected 10 times for grip strength and F-CPR, 7 times for knee extension muscle strength, 2 times for ankle dorsiflexion muscle strength and 3 times for proliferative retinopathy. In the ROC analyses, AUC, accuracy, sensitivity and specificity of the logistic regression were 0.75, 0.77, 0.52 and 0.82, respectively, and those of the random forest classifier were according to the average of 10 times and 10 cross validations 0.74 (SD = 0.01), 0.68 (SD = 0.02), 0.77 (SD = 0.04) and 0.66 (SD = 0.03), respectively.

**Table 4:** Multivariate association of fall-related variables with clinical parameters in stepwise logistic regression analysis.

Covariates	Fallers (Adjusted R^2^ = 0.200)	
	*Β*	*P*
Knee extension strength	− 0.698	0.002
Fasting C-peptide level	0.492	0.009
Dorsiflexion strength	− 0.432	0.047

Covariates: sex, fasting C-peptide levels, impaired vibratory sensation, presence of proliferative retinopathy, past history of stroke, taking sulfonylureas at discharge, skeletal muscle percentage, body fat percentage, knee extension strength, knee extension endurance, dorsiflexion strength, toe pinch force, grip strength, index of postural stability, and one-leg standing time.

**Table 5:** Average importance of covariates in the random forest model.

Covariates	Covariate importance
Grip strength	0.495
Fasting C-peptide level	0.397
Knee extension strength	0.058
Dorsiflexion strength	0.037
Presence of proliferative retinopathy	0.011

Covariates: sex, fasting C-peptide levels, impaired vibratory sensation, presence of proliferative retinopathy, past history of stroke, taking sulfonylureas at discharge, skeletal muscle percentage, body fat percentage, knee extension strength, knee extension endurance, dorsiflexion strength, toe pinch force, grip strength, and index of postural stability.

## Discussion

We conducted a follow-up questionnaire survey of falls that occurred during the first year after discharge from hospital in persons with type 2 diabetes and analyzed data using machine learning to investigate the relationship between various indices measured during hospitalization and falls after one year. There were three significant findings. First, 19% of the participants experienced falls within one year after discharge. Second, muscle strength-related variables were the most important predictors of falls according to both logistic regression and random forest classifier. Third, the F-CPR level was an important predictor of falls, and, to the best of our knowledge, is a novel finding in terms of risk factors for falls.

The annual rate of falls was reported to range from 17 to 32% in community-dwelling older adults^1,8,17,18^ and 22–40% in older adults with diabetes^10,11^. In the current study, 19% of participants experienced falls within one year after discharge, even though participants were younger with a median age of 62 years compared with previous reports. This indicates that early measures are needed to prevent falls in persons with type 2 diabetes.

In this study, compared with the non-faller group, the faller group had a significantly higher proportion of females and higher incidence of impaired vibratory sensation, proliferative retinopathy and stroke. F-CPR levels were also significantly higher and values for skeletal muscle percentages, muscle strength-related variables and balance capability-related variables were significantly lower compared with the non-faller group. To investigate the important risk factors for falls, we used logistic regression and random forest^19^. Most of the important variables were shared between these analyses, including knee extension strength, F-CPR level and dorsiflexion strength. These results differ from risks for falls shown in previous studies, which may have been influenced by differences in the explanatory variables, condition of study participants and the methods of analysis. Our study participants were younger and had better physical function than those in previous studies.

A meta-analysis showed that muscle weakness in the upper or lower extremities was associated with future falls^20^. Persons with type 2 diabetes, especially those with diabetic polyneuropathy, were shown to have decreased muscle mass and strength in lower limb extremities compared with non-diabetic control participants^21–23^. Although the current study did not have non-diabetic controls, participants had knee extension strength approximately 30% lower than middle-aged obese non-diabetic Japanese in a previous study^24^. Also shown was that muscle strength of knee extensors and the ankle plantar flexor in type 2 diabetic participants were about 30% lower than in healthy control participants^21^. In a cross-sectional study of aging and muscle function in a general population in Belgium, the mean knee extensor muscle strength in the group aged > 70 years was about 30% lower in men and 40% lower in women compared with the group aged 50–60 years^25^. These data suggest that lower body muscle strength in diabetic individuals declined faster than in healthy individuals, which is a major reason for the high prevalence of falls in diabetic individuals.

Grip strength and proliferative diabetic retinopathy were selected as important risk factors only in random forest. In particular, grip strength was the most important variable for falls in random forest, whereas knee extension strength was the most important variable in logistic regression. Since grip strength was reported to correlate with muscle mass or strength in various regions of the body^26,27^, as in the present study, certain predictive models may reflect the risk of falls more strongly than individual measures of lower extremity muscle strength, such as knee extension strength and dorsiflexion strength. Since poor vision is a risk factor for falls^28^, it is reasonable that proliferative retinopathy was selected by random forest in this study.

Unexpectedly, the F-CPR level was an important risk factor for falls with both logistic regression and the random forest classifier showing that F-CPR was among the most important risk factors for falls. Although those results cannot indicate a causal relationship, insulin resistance may be associated with falls. The Third National Health and Nutrition Examination Survey (NHANES III) showed that the elevated homeostasis model assessment of insulin resistance (HOMA-IR) was independently associated with lower SMI^29^. Furthermore, it was reported that abdominal circumference and metabolic syndrome, which are associated with insulin resistance, are associated with the risk of falls^30^. However, F-CPR levels were independent of lower limb muscle strength in this study. Since insulin resistance in skeletal muscle has been reported to inhibit skeletal muscle proliferation^31^, high serum F-CPR may be associated with lower future skeletal muscle mass and skeletal muscle strength. Another possibility regarding the link between falls and F-CPR is that insulin resistance increases the risk of falls by affecting cognitive function. Insulin resistance was shown to be associated with a cognitive decline in several prospective studies^32–35^. Decreased executive function was associated with the occurrence of falls^36^, which is impaired in persons with type 2 diabetes beginning in middle age.

In the current study, balance capability, diabetic polyneuropathy and insulin treatment were less important risk factors for falls than muscle strength and F-CPR.

Whether diabetic neuropathy and balance capability are independent risk factors for falls has not been concluded^10^. Because lower limb muscle strength, balance capability and peripheral neuropathy are interrelated, and because measurement methods and participants’ backgrounds vary among studies, it is difficult to assess the extent of the impact of these risk factors on falls in diabetic patients. However, the previous reports that showed a significant association between falls and DPN or balance capability did not assess lower extremity muscle strength and included participants with more severe DPN compared with studies that did not show such associations^9,10,37,38^. In addition, the reports that evaluated both balance capability and DPN showed that balance capability was a stronger risk factor for falls than DPN^10,11^. The participants in the current study may have had less severe DPN than those in the study that found that DPN was an independent risk factor for falls. Furthermore, since the median (25th percentile, 75th percentile) age of the current study population was 62 (49, 68) years, which is younger than in previous reports, it is possible that their balancing capabilities were better than those shown previously.

Insulin treatment has been shown to be an independent risk factor for falls, but in this study that was not the case. The Health ABC Study reported that insulin-treated individuals with HbA1c ≤ 6.0% were approximately 4.4 times more likely to fall than those without such treatment^10^. Hypoglycemia may be involved in the high incidence of falls in older adult diabetic patients on insulin therapy. Older adults are less aware of hypoglycemia and have a higher blood glucose threshold for impaired consciousness than younger persons. The current study participant population was comprised of a large number of patients younger than those in previous studies, making them less likely to be aware of hypoglycemia. In addition, the hospitalizations in the previous year may have resulted in fewer hypoglycemic incidents due to appropriate education and optimization of treatment.

In the current study, the AUCs of logistic regression and the random forest classifier were comparable. This result was in line with the results of previous studies, which showed that the accuracy of the predictive models did not change when comparing machine learning with traditional statistical methods^39–41^. However, in the current study, the random forest classifier newly selected "grip strength" and “retinopathy” as covariates, which were different from the results of logistic regression. This suggests that use of machine learning in addition to logistic regression can help identify new risk factors.

### Limitations of this study

As for limitations of the study, first, the tracking rate was not as high as desired. The follow-up rate of a cohort survey is usually required to be at least 80% whereas the current follow-up rate was 70%. This may have resulted in a selectivity bias. Second, since we mailed the questionnaire one year after discharge, recall bias may have been present. A previous study showed that 13% of participants with confirmed falls could not recall having a fall at the end of the study (12 months)^42^. Therefore, we may have underestimated the rate of falls. Third, the follow-up period was limited to one year. Extending the follow-up period might have shown increased rates of falls and uncovered new predictors of falls. Fourth, the number of participants in our study was too small to create a useful predictive model of falls. The logistic regression model considered main effects only to avoid potential overfitting due to the small sample size in relation to the number of possible combinations of predictors when interaction terms were included. We consider that the random forest model can capture such interactions effectively, and hence it is possible to investigate predictive risk factors from different perspectives by combining the two models, which have different mechanisms. Although our analysis may identify major risk factors, as similar variables were selected from both models, more detailed interaction effects should be investigated and validated using larger samples. Such future studies are needed to confirm our findings and to develop a useful predictive model for falls. Fifth, the default hyperparameter settings, which were used in random forest, may cause overfitting. However, we expected that, even if each decision tree overfitted the bootstrapped training dataset, the majority-voting with a sufficiently large ensemble can reduce the negative effect of the overfitting and bring out the predictive power of the decision trees. As n_estimators = 100 in the default setting are relatively large considering that the number of participants in this study was 226, we consider that overfitting may be avoided in the resultant prediction model. Moreover, using tenfold CV can lessen the possibility of overfitting. However, we cannot exclude the possibility that the results obtained in this study are overfitted for our participants. The results need to be validated in other cohorts. Sixth, study participants were hospitalized with poor glycemic control. Therefore, it is unclear whether the results can be extrapolated to patients with type 2 diabetes who were attending an outpatient clinic and were not recently hospitalized. Verification of the results requires a similar group who only attended outpatient clinic. Finally, the study included patients on insulin therapy, and serum F-CPR levels may be underestimated in these patients. Moreover, non-diabetic individuals were not included, and it is unclear whether the results of this study, especially that for F-CPR, can be applied to predict falls in non-diabetic individuals.

In conclusion, we investigated the frequency of falls and their risk factors 1 year after discharge from the hospital in patients with type 2 diabetes. The results showed that falls occurred in 19% of the participants, with knee extension strength, F-CPR and dorsiflexion strength selected as predictors of falls according to logistic regression analysis. Knee extension strength, grip strength, F-CPR and dorsiflexion strength were important predictors in the random forest analysis. We newly found that F-CPR was an important predictor of falls in persons with type 2 diabetes. Muscle weakness and insulin resistance may be strongly associated with falls in type 2 diabetic patients.

## Methods

### Study design and participants

We conducted a questionnaire survey on falls one year after discharge of 316 persons with type 2 diabetes who had been admitted to the University of Tsukuba Hospital for the treatment of diabetes. We also evaluated their physical capabilities during the hospitalization from February 2014 to May 2018. These study participants were completely independent in walking and activities of daily living. Exclusion criteria included: (1) vitreous hemorrhages or detached retina; (2) class II or higher New York Heart Association Functional Classification; (3) being treated for malignancies; (4) taking glucocorticoids, Cushing's syndrome or acromegaly; (5) post-gastrectomy; (6) unable to walk independently without assistive devices resulting from disorders of the hip or knee joints, paralysis or paresis caused by central nervous system disorders; (7) neuropathy due to causes other than diabetes and (8) difficulty in understanding instructions.

This study was approved by the Ethics Committee of the University of Tsukuba Hospital (H27-31) and conducted according to the Declaration of Helsinki. Written informed consent was obtained from all participants.

### Clinical data and laboratory tests

We collected socio-demographic information, medical history, anthropometric data and information on medications that participants took on admission and at discharge. Body composition was measured using bioelectrical impedance analysis (InBody 720, Biospace, Tokyo, Japan). SMI was calculated by dividing the limb skeletal muscle mass (kg) by the square of the height (m^2^)^43^. Diabetic retinopathy was evaluated by ophthalmologists. Ankle-brachial pressure index (ABI) and brachial-ankle pulse wave velocity (baPWV) were measured (BP-203RPE, Colin Medical Technology, Tokyo, Japan). Patients were classified as having peripheral artery disease (PAD) when the value for either of the lower limbs was less than 0.9. For baPWV, after measuring each side the value on the higher side was used. Cardiac autonomic nervous function was assessed by measuring the coefficient of variation of R-R intervals (CVR-R) at rest. Participants with CVR-R < 2.0% were classified as having cardiac autonomic neuropathy^44^.

Blood samples were collected in the morning after an overnight fast within 3 days after admission. Plasma glucose and serum total cholesterol, high-density lipoprotein-cholesterol (HDL-C), triglycerides (TG) and creatinine levels were determined using an automated analyzer (Hitachi High-Technologies, Tokyo, Japan). HbA1c was measured by high-performance liquid chromatography (Tosoh, Tokyo, Japan). Serum LDL-C levels were measured by a homogeneous assay (Sekisui Medical, Tokyo, Japan). Albumin excretion rate (AER) was measured using a turbidimetric immunoassay (Nittobo Medical, Tokyo, Japan). Diabetic nephropathy was defined as AER ≥ 30 mg/d. Estimated glomerular filtration rate (eGFR) was calculated using an equation modified for the Japanese: eGFR = 194 × sCre − 1.0949 × Age − 0.287 × 0.739 (if female)^45^. Serum C-peptide level was measured by an enzyme immunoassay (Tosoh, Tokyo, Japan).

### Follow-up of falls

Fall was defined as “coming in contact with the ground (or floor) from a standing or sitting position with a body part other than the foot in contact with the ground (floor surface) against the patient's intention”^46^. The reliability of a survey of the occurrence of falls over a previous one-year period using the recall method was confirmed^42,46^. A fall history for the previous year was obtained on hospital admission. One year after discharge, we mailed study participants a questionnaire asking about the number of falls (never, once or twice or more) experienced within that 1-year period.

### Diabetic polyneuropathy

Diabetic polyneuropathy (DPN) was diagnosed based on two or more of the following four criteria from The Diabetic Neuropathy Study Group in Japan^47^ and Michigan Neuropathy Screening Instrument ^48^: decreased vibration perception using a 128-Hz tuning fork at the bilateral medial malleoli (< 10 s); loss of tactile sensation using a 10 g monofilament at the bilateral foot; decreased or loss of bilateral Achilles jerk reflex; and numbness, pain, paresthesia or hypoesthesia in the bilateral lower limbs or feet.

### Physical activity

Physical activity during hospitalization was measured using an accelerometer (Mediwalk: Terumo Co., Tokyo, Japan). Information was collected on the average number of steps, average moderate-to-vigorous physical activity time (3 metabolic equivalents or more) and energy expenditure through exercise.

### Muscle strength

#### Knee extension strength and knee extension endurance

Measurements of knee extension strength and knee extension endurance were performed on the dominant foot side using a torque machine (Biodex System3: Sakai Medical, Tokyo, Japan). For evaluation of knee extension strength, the participant performed three consecutive knee extension operations with maximum effort in isokinetic muscle strength measurement (60°/s), and the maximum torque value (Nm/kg) was used as the representative measurement value. Knee extension endurance was determined by measuring the total work (J) from 20 continuous knee extensions with maximum effort by isokinetic muscle strength measurements (300°/s).

### Dorsiflexion strength of the ankle joint

Dorsiflexion strength of the ankle joint was measured using a hand-held dynamometer (Anima Co., Tokyo, Japan). The participant adjusted the height of the chair so that the angle between the knee joint and the ankle joint was 90° while being seated with the heel on the floor and the foot was raised toward the anterior shin. The joint was placed in the maximum dorsiflexion position, and preparations were made for measurement. The examiner applied the attachment of the hand-held dynamometer to the back of the participant’s foot and applied maximum pressure in the ankle plantar flexion direction to break the participant’s maximum ankle dorsiflexion. The test was performed twice on each side and the highest value for the right side and left side, respectively, was averaged.

### Toe pinch force

Toe pinch force was measured using a pinch force dynamometer (Checker-kun, Nisshin Sangyo Inc., Saitama, Japan). The participant sat on a chair with arms crossed over the chest. The dynamometer was attached to the foot between the great toe and second toe while the participant remained in a seated position with hip and knee joints at 90° of flexion^49^. The test was performed twice on the left and right side, respectively, and the best result for each side was averaged.

### Grip strength

Grip strength of the dominant hand was measured using a Smedley analog grip meter (ST100 T-1780, Toei Light Co., Tokyo, Japan). The maximum value (kgf) was taken as the measured value.

### Balance capability

Balance capabilities were assessed by the one-leg standing time with eyes open^50^ and the index of postural stability (IPS). IPS was measured using a gravicorder (GP-6000, Anima Co., Tokyo, Japan) as described elsewhere^51^. First, the participants stood in a resting position with the inside of the foot at a distance of 10 cm on the gravicorder to measure the instantaneous fluctuations in the center of pressure (COP) at a sampling frequency of 20 Hz. Then, participants were instructed to incline the body to the front, rear, right and left without bending the body and moving the feet. Instantaneous fluctuations in COP were measured at each position. IPS was calculated as “log [(area of stability limit + area of postural sway) / area of postural sway]”. Area of stability limit was calculated as the “front and rear center movement distance between anterior and posterior positions × the distance between right and left positions”. Area of postural sway was calculated as “average measurement value in 10 s under anterior, posterior, right, left and center positions”. The area of postural sway was calculated as the mean sway area of the 5 positions. In addition, IPS was measured while the participant stood with closed eyes on the gravicorder which was covered with foam rubber (AIREX Balance-pad Elite, Airex AG, Switzerland) as modified IPS (mIPS)^51^.

### Flexibility

Truncal flexibility was assessed by measuring the finger-floor distance (FFD). Participants standing on a 20 cm high platform flexed the trunk and dropped both arms toward the floor^52^. The length from the top of the platform to the tip of the third finger was measured as the FFD.

### Statistical analysis

The sample size for the study was determined by considering the number of cases in previous studies of falls^17^ and the number of participants that could be enrolled annually in our study. Study participants were those without missing data.

Continuous variables were checked for normal distribution using the Shapiro–Wilk test and were expressed as mean ± SD or median (25th percentile, 75th percentile) based on distribution. Unpaired t-test and Mann–Whitney’s U test were used for continuous variables with normal distribution and those with non-normal distribution, respectively. Categorical variables were presented as numbers (percentage) and analyzed using the chi-squared test. Classification analysis was performed based on the identified covariates that were significantly different between faller and non-faller groups. In addition, we used covariates that were not significantly different between our study groups but have been reported as risks for falls in previous studies^5,53,54^. We applied two types of classification models, that is, logistic regression and a random forest classifier, to investigate relationships between fall risk and clinical parameters from different perspectives. These methods were chosen because of their capabilities to quantitatively assess the importance of variables included in the models to predict falls. Models were fit with a stepwise variable selection procedure where the initial variable subset is set as empty and explanatory covariates in the models are sequentially added to or removed from the current subset by evaluating predefined criteria. In logistic regression, we fit the model to the whole dataset and selected the coefficients with *p* < 0.05 in terms of the likelihood ratio test. We kept the variable with the smallest *p* value in each step. The performance of the random forest classifier was evaluated by tenfold cross validation. Specifically, the dataset was split into training (9/10) and test (1/10) partitions. The model was fit to the training partition and subsequently tested by the test partition. This procedure was repeated for all folds and the performance was calculated. The entire procedure was repeated 10 times, and the averaged performance was reported (10-times tenfold cross validation). In each cross validation, the variable subsets with the best estimated predictive performance regarding accuracy were identified via the stepwise selection procedure. We also reported the statistics of the selected subsets. The random forest hyperparameters in this study were the default values of the library. To compare the accuracy of the predictive ability of logistic regression and the random forest classifier, receiver operating characteristic (ROC) analysis was performed for each, and accuracy, sensitivity, specificity and the area under the curve (AUC) were determined.

For analysis, Scipy based on Python (programming language) was used, R (GLM) for logistic regression, and imbalanced-learn for the random forest classifier. The criterion for determining statistical significance was 5%.
